# Use of colony-based bacterial strain typing for tracking the fate of *Lactobacillus *strains during human consumption

**DOI:** 10.1186/1471-2180-9-251

**Published:** 2009-12-07

**Authors:** Eshwar Mahenthiralingam, Angela Marchbank, Pavel Drevinek, Iveta Garaiova, Sue Plummer

**Affiliations:** 1 Cardiff School of Biosciences, Cardiff University, Cardiff, Wales, CF10 3TL, UK; 2 Paediatric Department, 2nd Medical School, V Uvalu 84, 150 06 Prague, Czech Republic; 3 Obsidian Research Ltd., Unit 2 Christchurch Road, Baglan Industrial Park, Port Talbot, SA12 7BZ, Wales, UK

## Abstract

**Background:**

The Lactic Acid Bacteria (LAB) are important components of the healthy gut flora and have been used extensively as probiotics. Understanding the cultivable diversity of LAB before and after probiotic administration, and being able to track the fate of administered probiotic isolates during feeding are important parameters to consider in the design of clinical trials to assess probiotic efficacy. Several methods may be used to identify bacteria at the strain level, however, PCR-based methods such as Random Amplified Polymorphic DNA (RAPD) are particularly suited to rapid analysis. We examined the cultivable diversity of LAB in the human gut before and after feeding with two *Lactobacillus *strains, and also tracked the fate of these two administered strains using a RAPD technique.

**Results:**

A RAPD typing scheme was developed to genetically type LAB isolates from a wide range of species, and optimised for direct application to bacterial colony growth. A high-throughput strategy for fingerprinting the cultivable diversity of human faeces was developed and used to determine: (i) the initial cultivable LAB strain diversity in the human gut, and (ii) the fate of two *Lactobacillus *strains (*Lactobacillus salivarius *NCIMB 30211 and *Lactobacillus acidophilus *NCIMB 30156) contained within a capsule that was administered in a small-scale human feeding study. The *L. salivarius *strain was not cultivated from the faeces of any of the 12 volunteers prior to capsule administration, but appeared post-feeding in four. Strains matching the *L. acidophilus *NCIMB 30156 feeding strain were found in the faeces of three volunteers prior to consumption; after taking the *Lactobacillus *capsule, 10 of the 12 volunteers were culture positive for this strain. The appearance of both *Lactobacillus *strains during capsule consumption was statistically significant (p < 0.05).

**Conclusion:**

We have shown that genetic strain typing of the cultivable human gut microbiota can be evaluated using a high throughput RAPD technique based on single bacterial colonies. Validation of this strategy paves the way for future systematic studies on the fate and efficacy of bacterial probiotics during human clinical trials.

## Background

The application of bacterial probiotics or nutritional supplements containing these microorganisms represents one of the fastest growing areas in both industrial/clinical microbiology. Probiotics have been defined by the World Health Organisation live microorganisms which when administered in adequate amounts, confer health benefits on the host [1,2]. The Lactic Acid Bacteria (LAB; including the genera *Lactobacillus*, *Enterococcus *and *Streptococcus*) comprise the most commonly used probiotics and have been shown to have therapeutic or prophylactic potential for a number of human and animal dietary conditions or diseases [1,3,4]. The natural diversity of LAB in the human gut has been studied by cultivation dependent methods and conventional phenotypic identification of constituent species. More recently, powerful cultivation-independent methods such as microbial metagenomics have begun to shed light on the total microbial diversity of human gut [5]. Although metagenomic studies allow detailed analysis of what species of bacteria are present, currently they provide only limited information on the level of strain diversity that may occur for any given LAB species.

Characterisation of the strain diversity of LAB species has only really begun in the last decade. Yeung et al[6] successfully used macrorestriction and Pulsed Field Gel Electrophoresis (PFGE) to examine the genotypic diversity of probiotic lactobacilli and showed that several commercial probiotic formulations contained the same bacterial strain. Vancanneyt et al. [7] used a combination of Amplified Fragment Length Polymorphism (AFLP) and PFGE to specifically examine *Lactobacillus rhamnosus *species probiotics and also demonstrated the presence of multiple indistinguishable strain types present in a variety of probiotic products. PCR-fingerprinting methods analysis have also been used to examine the strain diversity of *Lactobacillus *probiotics. For example, Schillinger et al. [8] used Random Amplified Polymorphic DNA (RAPD) analysis to differentiate *Lactobacillus *strains cultivated from probiotic yogurts. Pena et al[9] used Repetitive Element PCR (REP) profiling to examine the genetic diversity of intestinal *Lactobacillus *species colonising different transgenic mouse-lines; they demonstrated that mice with colitis due to IL-10 deficiency were colonised with a different population of strains in comparison to those without colitis. Multilocus sequence typing, a very powerful nucleotide sequence based strain differentiation methods has also been recently developed for *Lactobacillus plantarum *[10] and *Lactobacillus casei *[11]. However, genetic typing methods that work at the strain level have seen limited use in their direct application to the human gut microbiota and have not yet been applied to specifically track the fate of a specific probiotic strain during consumption.

Understanding the dynamics of gut colonisation by bacterial probiotics is an important parameter for the future clinical development of these therapeutic agents. We set out to determine if individual *Lactobacillus *species strains could be tracked after human consumption of the encapsulated bacteria. RAPD was selected as a suitable strain typing method to answer this question because: (i) as a PCR-based method it was amenable to high throughput, and, (ii) we knew from past-experience that if the RAPD method was systematically developed to target specific bacterial species, then its discriminatory power can be comparable to state-of-the-art DNA sequence-based genotyping methods such as multilocus sequence typing [12]. Here we describe the systematic development of a RAPD fingerprinting method for a broad range of LAB species and its optimization to allow direct application to single bacterial colonies. Using this novel high throughput colony strain typing strategy we were then able for the first time to track the fate of specific *Lactobacillus *strains after their consumption by human volunteers.

## Results

### Development of a RAPD fingerprinting method for Lactic Acid Bacteria

**Figure 1:**
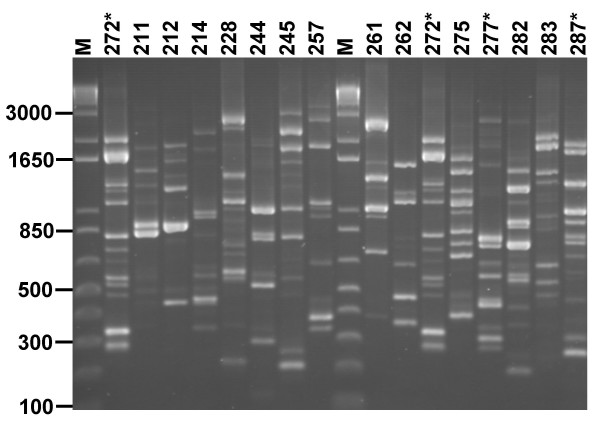
**Useful RAPD primers producing diverse polymorphisms from *L. acidophilus***. The fingerprint patterns generated from strain LMG 9433^T ^are shown for 15 of the primers which were capable of amplifying diverse polymorphisms. The primer number is shown above each lane (the corresponding primer sequence is given in Table 2) and the size of relevant molecular markers (lane M) indicated in bp. The primers selected for typing of LAB are shown (*) with primer 272 being run in duplicate as a control and test.

To systematically develop a RAPD typing scheme for LAB species, a set of 100 RAPD primers which had proven successful for strain typing other bacterial species [13,14] were screened for their ability to amplify multiple polymorphisms from *L. acidophilus*. Fifteen primers (Table 1) were found to reproducibly amplify 8 or more random DNA fragments from the reference strain *L. acidophilus *LMG 9433^T ^that ranged in size from 200 to 4000 bp (Fig. 1). The complexity of these profiles indicated that discriminatory typing of LAB isolates with these primers was possible.

**Table 1:** Specifications of useful RAPD primers for typing Lactic Acid Bacteria

Primer name:	Sequence (5' to 3')	Approximate No. of polymorphisms from *L. acidophilus *LMG 9433^T^
272	AGCGGGCCAA	13

277	AGGAAGGTGC	13

287	CGAACGGCGG	12

211	GAAGCGCGAT	11

275	CCGGGCAAGC	11

282	GGGAAAGCAG	11

244	CAGCCAACCG	10

245	CGCGTGCAAG	10

257	CGTCACCGTT	9

283	CGGCCACCGT	9

212	GCTGCGTGAC	8

214	CATGTGCTTG	8

228	GCTGGGCCGA	8

261	CTGGCGTGAC	8

262	CGCCCCCAGT	8

The primers with the most diverse polymorphisms, 272, 277 and 287 (Table 1; Fig. 1) were selected for genotyping isolates of further LAB species beyond *L. acidophilus*. Primary typing was performed with primer 272 because of its known discriminatory power [13,14], and secondary confirmation of strain type was performed with primers 277 and 287.

### LAB isolates examined

**Figure 2:**
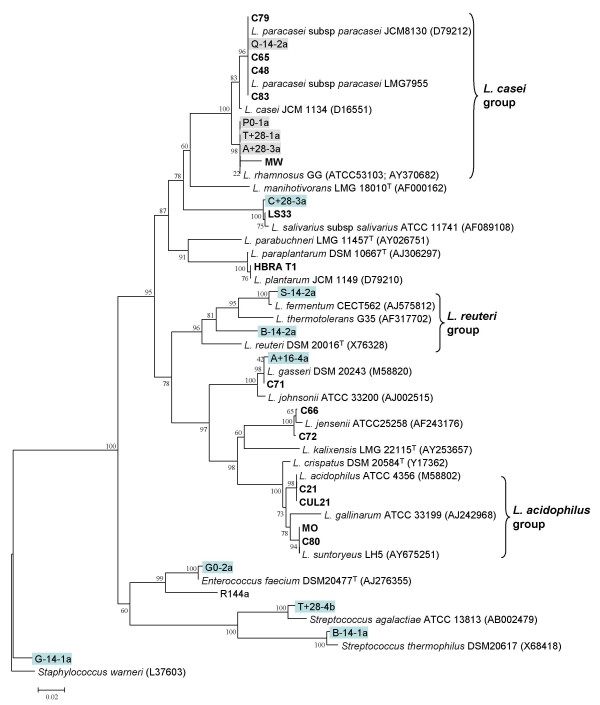
**Phylogenetic distribution of LAB probiotics and bacteria cultivated during the feeding study**. A phylogenetic tree of aligned 16S rRNA genes from representative *Lactobacillus *reference strains, commercial probiotic strains and dominant isolates recovered during the feeding trial is shown. Probiotic strains are shown in bold font and isolates from the feeding study are highlighted by the grey boxes. The tree was rooted with the 16S rRNA gene from *Staphylococcus warneri *ATCC 27836 and the genetic distance scale and bootstrap values indicated.

A collection of 38 LAB isolates was assembled to assess the discriminatory power of the RAPD fingerprinting method (Table 2). The collection comprised reference isolates and Type strains of known LAB species obtained from recognised culture collections (14 isolates, 9 species; Table 2). In addition, commercially marketed probiotic products were purchased and their constituent LAB isolates cultured and purified (24 isolates, 11 species; Table 2). Previous studies have shown that the speciation and labelling of commercially marketed probiotics may often be inaccurate [15,16]. Therefore prior to examining the ability of RAPD to differentiate LAB isolates, sequence and phylogenetic analysis of the 16S rRNA gene was used to systematically identify the species of all LAB isolates cultured from commercial samples (Fig. 2; Table 2). To test the accuracy of this speciation strategy, control sequences from *L. brevis *LMG 6906^T ^and *L. johnsonii *LMG 9436^T^were obtained and found to cluster appropriately with the published sequences from these Type strains (data not shown). The majority of the cultivable bacteria contained within the commercial probiotic products were found to belong to the *L. casei *group (*L. casei*, *L. paracasei *and *L. rhamnosus*; 9 isolates) and *L. acidophilus *group (*L. acidophilus*, *L. gallinarum *and *L. suntoryeus *species; 6 isolates) (Fig. 2; Table 2). Other LAB species identified included (Table 2): *L. gasseri *(3 isolates), *L. jensenii *(2 isolates), *Enterococcus faecalis *(2 isolates), and *L. salivarius*, *L. plantarum*, and *Pediococcus pentosaceus *(single isolates, respectively).

**Table 2:** Reference, probiotic and faecal LAB isolates examined or isolated during the study

Isolate name (partial 16S rRNA gene sequence Accession no.)	Species or 16S rRNA gene closest BLAST match (Accession no. of closest match)	Source or product from which isolate was cultivated	RAPD strain type
**Reference isolates**			

LMG 11428	*L. acidophilus*	Rat faeces	1

LMG 11430	*L. acidophilus*	Human	1

LMG 11467	*L. acidophilus*	Human	1

LMG 11469	*L. acidophilus*	Rat intestine	1

LMG 8151	*L. acidophilus*	Acidophilus milk	1

LMG 9433^T^	*L. acidophilus*	Human	1

LMG 6906^T^	*L. brevis*	Human faeces	9

LMG 6904^T^	*L. casei*	Cheese	10

LMG 6901^T^	*L. delbruecki subsp. bulgaricus*	Yogurt	13

LMG 9203^T^	*L. gasseri*	Human	14

LMG 9436^T^	*L. johnsonii*	Human blood	15

LMG 6907^T^	*L. plantarum*	Pickled cabbage	19

LMG 7955 (EF442275)	*L. paracasei subsp. paracasei*	-	16

ATCC 29212 (EF442298)	*Enterococcus faecalis*	Human urine	26

**Probiotic and commercial isolates**			

NCIMB 30156 (CulT2; EF442276)	*L. acidophilus *(NCFM; CP000033)	Cultech Ltd.	1

C21 (EF442277)	*L. acidophilus *(NCFM; CP000033)	Commercial^a^	1

C46 (EF442278)	*L. acidophilus *(NCFM; CP000033)	Commercial^a^	1

HBAP T1 (EF442279)	*L. acidophilus *NCFM (CP000033)	Commercial probiotic^b^	1

C80 (EF442280)	*L. suntoryeus *strain LH5 (AY675251)	Commercial^a^	3

MO (EF442281)	*L. suntoryeus *strain LH5 (AY675251)	Commercial probiotic^b^	3

BF T1 (EF442282)	*L. casei *subsp. *casei *ATCC 393 (AY196978)	Commercial probiotic^b^	10

C48 (EF442283)	*L. paracasei *subsp. *paracasei *DJ1 (DQ462440)	Cultech Ltd.	11

C65 (EF442284)	*L. paracasei *subsp. *paracasei *DJ1 (DQ462440)	Commercial^a^	12

C79 (EF442285)	*L. paracasei *subsp. *paracasei *DJ1 (DQ462440)	Commercial^a^	18

C83 (EF442286)	*L. paracasei *subsp. *paracasei *DJ1 (DQ462440)	Commercial^a^	17

P7 T1 (EF442287)	*L. paracasei *subsp. *paracasei *DJ1 (DQ462440)	Commercial^a^	21

GG	*L. rhamnosus *LR2 (AY675254)	Commercial probiotic^b^	27

FMD T2 (EF442288)	*L. rhamnosus *LR2 (AY675254)	Commercial probiotic^b^	20

MW (EF442289)	*L. rhamnosus *LR2 (AY675254)	Commercial probiotic^b^	20

C44 (EF442290)	*L. gasseri *TSK V1-1 (AY190611)	Cultech Ltd.	2

C71 (EF442291)	*L. gasseri *TSK V1-1 (AY190611)	Cultech Ltd.	7

SSMB (EF442292)	*L. gasseri *TSK V1-1 (AY190611)	Commercial probiotic^b^	22

C66 (EF442293)	*L. jensenii *KC36b (AF243159)	Cultech Ltd.	5

C72 (EF442294)	*L. jensenii *KC36b (AF243159)	Cultech Ltd.	4

NCIMB 30211 (CulT1; EF442295)	*L. salivaruis *subsp. *salivarius *UCC118 (CP000233)	Commercial^a^	25

HBRA T1 (EF442296)	*L. plantarum *strain WCFS1 (AY935261)	Commercial probiotic^b^	23

HBRA T3 (EF442297)	*Pediococcus pentosaceus *ATCC 25745 (CP000422)	Commercial probiotic^b^	24

C22 (EF442299)	*Enterococcus faecalis *NT-10 (EF183510)	Cultech Ltd.	8

**Faecal isolates from human probiotic feeding study**			

A+16-4a (EF442300)	*L. gasseri *TSK V1-1 (AY190611)	This study	28

A+28-3a (EF442301)	*L. rhamnosus *LR2 (AY675254)	This study	29

A+28-3b (EF442302)	*L. rhamnosus *LR2 (AY675254)	This study	29

B-14-1a (EF442303)	*Streptococcus salivarius *ATCC 7073 (AY188352)	This study	31

B-14-2a (EF442304)	*L. mucosae *BJ18-2 (AY341550)	This study	32

B-14-4a (EF442305)	*Streptococcus salivarius *ATCC 7073 (AY188352)	This study	33

B-14-4b (EF442306)	*Streptococcus salivarius *ATCC 7073 (AY188352)	This study	34

B0-3a (EF442307)	*Streptococcus salivarius *ATCC 13419 (M58839)	This study	30

C-14-4b (EF442308)	*Enterococcus faecalis *ABPL 007 (DQ983196)	This study	35

C+28-3a (EF442309)	*L. salivaruis *subsp. *salivarius *UCC118 (CP000233)	This study	36

F-14-3a (EF442310)	*Enterococcus gallinarum *F02025 (DQ465366)	This study	38

G-14-1a (EF44211)	*Staphylococcus lugdunensis *ATCC 43809 (AB009941)	This study	40

G0-2a (EF44212)	*Enterococcus sanguinicola *BAA-781	This study	39

P-14-2a (EF44213)	*Enterococcus gallinarum *F02025 (DQ465366)	This study	43

P0-1a (EF44214)	*L. rhamnosus *LR2 (AY675254)	This study	41

P0-1b (EF44215)	*L. rhamnosus *LR2 (AY675254)	This study	41

P0-2a (EF44216)	*Staphylococcus *sp. CNJ924 PL04 (DQ448767)	This study	42

P+28-2a (EF44217)	*Staphylococcus warneri *PB1 (AY186059)	This study	44

Q-14-2a (EF44218)	*L. paracasei *subsp. *paracasei *DJ1 (DQ462440)	This study	47

Q-14-4a (EF44219)	*Streptococcus salivarius *clone (AM157451)	This study	48

Q0-1a (EF44220)	*Enterococcus faecalis *ABPL 007 (DQ983196)	This study	45

Q0-4a (EF44221)	*Staphylococcus *sp. CNJ924 PL04 (DQ448767)	This study	46

Q+28-2a (EF44222)	*Streptococcus *sp. clone (EF151147)	This study	49

R-14-4a (EF44223)	*Enterococcus faecalis *ABPL 007 (DQ983196)	This study	51

R-14-5a (EF44224)	*Enterococcus faecalis *ABPL 007 (DQ983196)	This study	52

R0-1b (EF44225)	*Weissella cibaria *ACA-DC 3411t2 (AJ422031)	This study	50

S-14-2a (EF44226)	*L. fermentum *strain L18 (DQ523484)	This study	53

T+28-1a (EF44227)	*L. rhamnosus *LR2 (AY675254)	This study	41

T+28-4b (EF44228)	*Streptococcus agalactiae *A909 (CP000114)	This study	54

^a ^Strain widely used in commercial applications however specific original source was not known

^b ^Strain cultivated from a commercially marketed probiotic formulation

### Testing the discriminatory power of the RAPD method on other LAB species

The broad collection of systematically identified LAB isolates (Table 2) were used to test the efficacy of the RAPD typing scheme. The reproducibility of the RAPD method was excellent, with all 14 reference strains demonstrating identical fingerprint profiles after duplicate analysis. In addition *L. acidophilus *LMG 9433^T ^was analysed by RAPD at multiple points throughout the study as an internal control; the same fingerprint profile was obtained on each occasion demonstrating that the LAB PCR genotyping scheme demonstrated the same high reproducibility as had been observed with previous RAPD studies on other bacterial species [13,14].

**Figure 3:**
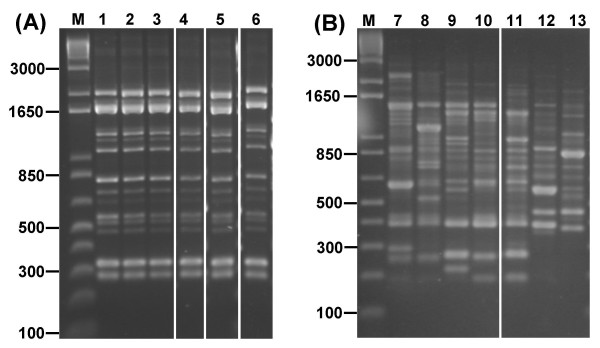
**Discrimination of LAB by RAPD typing**. The ability of PCR fingerprinting (with primer 272) to cluster identical isolates (Panel A) and differentiate distinct isolates within the *L. casei *group (Panel B) is shown. Strains shown in each lane are as follows: Panel A; 1, *L. acidophilus *LMG 9433^T^; lanes 2 to 6, matching *L. acidophilus *isolates LMG 11428, LMG 11430, C21, C46 and NCIMB 30211, respectively; Panel B; lanes 7 to 11, *L. paracasei *subsp *paracasei *isolates C48, C65, C83, C79 and LMG 7955, respectively; 12, *L. casei *LMG 6904 ^T^; and 13, *L. rhamnosus *MW. Molecular size markers were run in lane M and the size of relevant bands is indicated; panel A and B represent composite lanes taken from a single gel in each case.

RAPD fingerprinting was able to cluster genetically identical strains as well as differentiate distinct strains within closely related LAB species. For example, multiple isolates of *L. acidophilus *were found to possess identical RAPD fingerprints (using primer 272) to the type strain for the species, LMG 9433^T ^(Fig. 3, panel A). These included 4 additional reference isolates that had originally been recovered from diverse sources such as from rat and human faeces, as well as 4 isolates used in the commercial probiotic products (Table 2). All *L. acidophilus *isolates were genotypically indistinguishable even when examined with additional RAPD primers 277 and 287. These data suggested there was little genetic heterogeneity among isolates of *L. acidophilus *examined in this study. In addition they show that isolates genotypically identical to the *L. acidophilus *Type strain have been widely adopted for commercial use (Fig. 3, panel A; Table 2). Of the remaining 8 LAB reference isolates examined, 8 distinct RAPD strain types were found that corresponded to each LAB species (Table 2).

RAPD fingerprinting was also able to differentiate genetically unique strain types within very closely related species such as those within the *L. casei *group (Fig. 2); these included *L. casei*, *L. paracasei *and *L. rhamnosus *(Fig. 3, panel B). From this closely related complex of species (Fig. 2), a total of 9 distinct RAPD types (10, 11, 12, 16, 17, 18, 20, 21, and 27; Table 2) were identified. Two commercially marketed probiotics were found to contain the same strain of *L. rhamnosus *(isolates FMD T2 and MW, RAPD type 10; Table 2). Another commercial probiotic formulation contained an *L. casei *strain, designated BF T1, that was identical by RAPD to the *L. casei *Type strain LMG 6904^T ^(Table 2). Overall, the RAPD fingerprinting method was highly effective, working on all 38 LAB isolates examined irrespective of their species and reproducibly defining 26 RAPD types within this diverse collection (Table 2).

### Application of RAPD fingerprinting to single colonies

**Figure 4:**
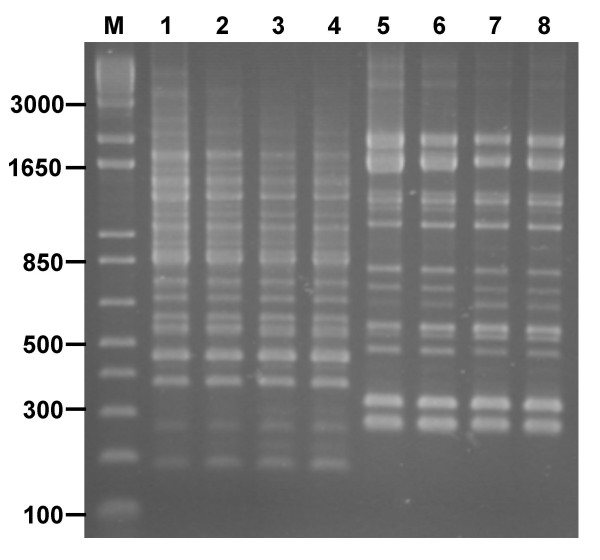
**Reproducibility of single colony RAPD fingerprints**. The polymorphismsamplified by primer 272 from conventionally extracted DNA compared to single colony Chelex^® ^extracted DNA are shown for two LAB strains as follows: lane 1, *L. rhamnosus *strain MW standard DNA extraction; lanes 2 to 4, single colonies of strain MW that were picked into Chelex^® ^resin, stored frozen and then extracted immediately prior to PCR; lane 5, *L. acidophilus *strain LMG 8151 standard DNA extraction; lanes 6 to 8, single colonies of strain LMG 8151 that were processed with Chelex^® ^as described. The size of relevant molecular size markers (lane M) are shown in bp.

To facilitate high throughput typing that could be applied to screening LAB isolates cultivated directly from human faeces, we evaluated if the PCR-fingerprinting method could be adapted for use on single bacteria colonies. Single colonies were picked with a sterile plastic tip and rapid boiling/cooling in a Chelex^® ^resin extraction buffer used to obtain DNA for PCR (see Methods). The RAPD fingerprints obtained from colonies processed in this way were identical to those produced from conventionally extracted high molecular weight DNA (Fig. 4). However, it was found that consistent profiles were only obtained if the RAPD PCR was set up immediately after the boiling and chilling cycles of the colony extraction procedure. The amplified PCR fingerprints deteriorated after subsequent frozen storage of the Chelex^® ^resin extracted DNA. To overcome this potential problem, we examined if prolonged frozen storage (-20°C) of the resuspended colony in Chelex^® ^resin prior to full extraction by boiling was possible. This procedure did not affect the quality of the RAPD profiles (Fig. 4). The ability to fingerprint from frozen stored colony material provided a high throughput strategy that could be used to systematically screen the multiple colony types isolated from human faeces as part of a *Lactobacillus *strain feeding study (see below).

### *Lactobacillus *species feeding study design

A small scale proof-of-principle human feeding study was performed to evaluate if the colony-fingerprint strategy could be used to track specific LAB strains from ingestion as capsule recovery from faeces. A capsule for oral administration was formulated to commercial standards which contained two *Lactobacillus *species isolates: *L. salivarius *strain NCIMB 30211 (1.8 × 10^10 ^colony forming units [cfu] per capsule) and *L. acidophilus *strain NCIMB 30156 (5.6 × 10^9 ^mean cfu per capsule). Twelve volunteers participated in a feeding study where the capsule was taken daily for 14 days; faecal samples were provided on days before, during and after consumption as described in the Methods. The volunteers were not advised to change their diets in any way other than to take the capsule once a day with some food on each of the trial days. At each faecal sampling point, LAB were plated as described below, enumerated and multiple colonies genotyped by RAPD.

### Cultivation of LAB species from human faeces

Although MRS agar is a well established cultivation medium for semi-selective culture of LAB species [17], we found that several non-LAB species, in particular Gram negative enteric bacteria were frequently encountered as contaminants after plating of human faeces (data not shown). To assist with selection of the *Lactobacillus *species in the feeding study, we investigated whether the addition of polymyxin B to MRS medium (MRS-P agar, see Methods) would increase the selectivity of this medium by acting as a counter-selection against coliforms. Addition of polymyxin B at a concentration of 120 units per ml of agar did not inhibit the viability of any of reference LAB species isolates (Table 2) or the two *Lactobacillus *strains incorporated into the capsule. However, MRS-P was highly effective at reducing the number of contaminating Gram negative enteric colonies seen after plating of human faeces.

To examine the efficacy of the semi-selective MRS-P developed for enrichment of the LAB species within faeces, 29 of the most dominant cultivable isolates recovered from 10 of the volunteers at days -14, 0 and 28 (before and after *Lactobacillus *feeding) were randomly selected for molecular identification. Using 16S rRNA gene sequence analysis these dominant isolates were identified as (Table 2; Fig 2): *Lactobacillus *species (10 isolates), *Streptococcus *species (7 isolates), *Enterococcus *species (7 isolates), *Weissella *species (1 isolate) and *Staphylococcus *species (4 isolates). The latter *Staphylococcus *isolates were the only non-LAB species isolated in high numbers on MRS-P agar after faecal plating. These data indicated that the MRS-P agar was effective for selection of LAB species after faecal culture.

### Tracking *Lactobacillus *strains after oral administration

**Figure 5:**
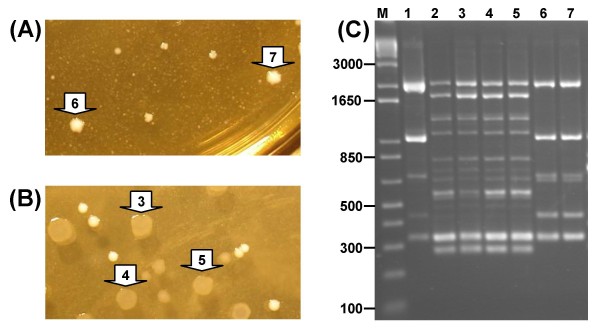
**Detection of *L. salivarius *and *L. acidophilus *strains after feeding**. The colony growth after plating of the day 7 faecal sample from volunteer F are show for the neat and third serial dilutions on MRS-P agar (panels A and B, respectively). Colonies picked for PCR fingerprinting are shown by the numbered arrows. The subsequent RAPD typing analysis is shown in panel C with the lane numbers corresponding to the colony numbers. Other lanes for panel C are as follows: M, molecular size markers (size in bp indicated); 1, *L. salivarius *NCIMB 30211 control and 2, *L. acidophilus *NCIMB 30156 control.

RAPD fingerprinting of the major colony morphotypes appearing after cultivation of each faecal sample was used to determine if the *Lactobacillus *strains had survived gastric and intestinal passage (Fig. 5). The mean faecal LAB count was 8.8 ± 2.7 × 10^6 ^cfu per g faeces when all volunteer samples were analysed; consumption of the lactobacilli did not significantly alter the total faecal LAB counts obtained from any of the volunteers (data not shown). Prior to the start of the study, *L. salivarius *strain NCIMB 30211, was not detected in any of the volunteers, however, strains matching *L. acidophilus *NCIMB 30156 were cultivated from three of the volunteers at the pre-feeding stage (Table 3). The appearance of this *L. acidophilus *(RAPD strain type 1; Table 2) at this point in the study was not unreasonable since it appeared to be a strain commonly found in food/probiotic products which may have been consumed by the volunteers (Table 2).

**Table 3:** Detection of *Lactobacillus *capsule strains and other faecal bacteria during the feeding study

Volunteer	Detection of strain in faecal samples before and after consumption of the *Lactobacillus *capsule^a^				Other recurrent strains^b^ (strains listed in Table 2)
		
	*L. salivarius *NCIMB 30211		*L. acidophilus *NCIMB 30156		
		
	Before	After	Before	After	
A^c^	-	-	-	+ (D7,21,28)	5 strains (*L. rhamnosus *A+28)

B^d^	-	+ (D2)	-	+ (D2)	2 strains (*S. salivarius *B0-3a)

C	-	-	+ (D-14)	+ (D16)	5 strains (*E. faecalis *C-14-4b; *L. salivarius *C+28-3a)

F^e^	-	+ (D7)	-	+ (D7)	1 strain (*E. gallinarum *F-14-3a)

G	-	+ (D2)	-	+ (D2)	4 strains (*S. lugdenensis *G-14-1a; *E. sanguinicola *G0-2a)

J^f^	-	-	-	+ (D12)	3 strains

N	-	-	+ (D-14, 0)	+ (D2,21,28)	2 strains (*L. acidophilus *NCIMB 30211)

P	-	-	-	+ (D7)	6 strains (*L. rhamnosus *P0-1a/n; *E. gallinarum *P-14-2a; *Staphylococcus *sp P0-2a; *S. warneri *P+28-2a)

Q	-	-	-	-	6 strains (*E. faecalis *Q0-1a; *Staphylococcus *sp Q0-4a; *Streptococcus *sp Q+28-2a)

R^g^	-	-	+ (D-14)	+ (D8)	5 strains (*E. faecalis *R-14-4a and R-14-5a; *W. cibaria *R0-1b)

S	-	+ (D2,7,21, 28)	-	+ (D7,21,28)	5 strains (*L. fermentum *S-14-2a)

T	-	-	-	-	3 strains (*L. rhamnosus *T+28-1a; *S. agalactiae *T+28-4b)

^a ^D = day of faecal sample

^b ^Recurrent strains cultivated from faecal sample provided at two or more time points

^c ^Day +14 sample from this volunteer was provided on day 16

^d ^Volunteer withdrew from the study on day 2

^e ^Volunteer withdrew from the study on day 7

^f ^Volunteer withdrew from the study on day 12

^g ^Volunteer withdrew from the study on day 8

After consumption of the capsule, the *L. salivarius *NCIMB 30211 strain was detected on day 2 in three volunteers (B, G and S), on day 7 in two volunteers (F, see Fig. 5; S), with only volunteer S remaining faeces positive for this strain on days 21 and 28 (7 and 14 days, respectively, after feeding stopped; Table 3). Increased detection of the *L. acidophilus *NCIMB 30156 strain was also seen with 10 of the volunteers culture positive for this strain at one or more sample points during the feeding period (volunteers A-C, F, G, J, N, P, R and S), and 3 of these (A, N, and S) remained positive on days 21 and 28 (Table 3). *L. salivarius *NCIMB 30211 was never the dominant cultivable LAB strain and was detected at 10^2 ^to 10^4 ^per g faeces (Fig. 5). In contrast, *L. acidophilus *NCIMB 30156 was the most dominant colony morphotype in volunteers A (day 7 and 28), B (day 2), F (day 7; see Fig. 5) and N (day 2, 21 and 28; Table 3), where it represented 38% or greater of the total LAB count. The mean LAB count for these volunteers at these time points was 1.8 ± 7.6 × 10^7 ^per g faeces indicating that *L. acidophilus *NCIMB 30156 must have been present at a level of at least 10^7 ^per g of faeces.

Statistical evaluation of *Lactobacillus *feeding in terms of gut colonisation was carried out assuming a null hypothesis that: "Consumption will lead to the subsequent detection by cultivation of the constituent strains within the capsule in the faeces of each subject." Chi Squared analysis demonstrated that the distribution of *L. salivarius *NCIMB 30211 was significant, with none of the volunteers being positive prior to feeding, and 4 being culture positive (B, F, G and S; Table 3) at least once during the feeding period of the trial (Chi square = 4.8; p < 0.05). The distribution of *L. acidophilus *strain NCIMB 30156 was also significant (3 positive prior to feeding and 10 culture positive during feeding, Table 3; Chi square = 8.2, p < 0.01), suggesting that consumption of the organism had led to a significant increase in gut carriage of this *L. acidophilus *strain. However, limited persistence of the strains was observed in the culture positive volunteers after feeding ceased. For *L. acidophilus *NCIMB 30156, 10 volunteers were culture positive at least once during the feeding period, this fell to 3 who were still positive on day 21 and 28 (Table 3). With *L. salivarius *NCIMB 30211 only volunteer S retained the strain in faeces at day 21 and 28 after consumption had ceased (Table 3).

### Specific LAB strains persist in individual humans

**Figure 6:**
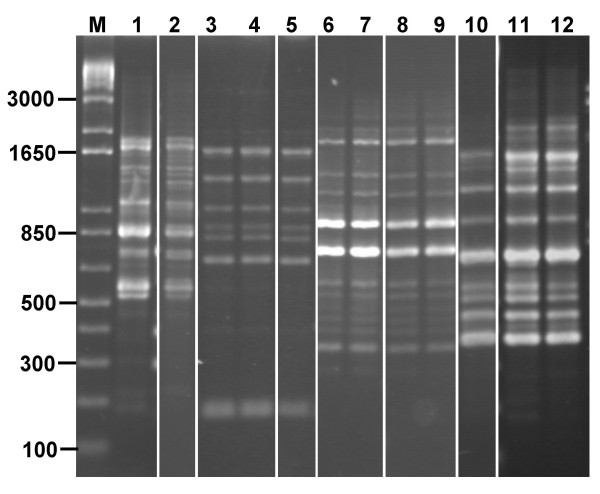
**Recurrent LAB strains carried by the human volunteers**. Several different strains of LAB were cultivated at several sampling points during the *Lactobacillus *feeding trial. RAPD fingerprints of these persistent strains are shown for the following in each lane: 1, *L. rhamnosus *A+7-5a; 2, A+28-3b*; 3, *E. sanguinicola *G0-2a*; 4, G0-2b; 5, G+21-1a; 6, E. faecalis Q0-1a; 7, Q0-1b; 8, Q+28-1a, 9, Q+28-1b; 10, *L. rhamnosus *T0-2a; 11, T+23-1a; 12, T+28-1b (systematic identification for the latter strains shown in Table 2). Molecular size markers are shown in lane M (size in bp indicated) and the figure is a composite of lanes drawn from 8 gels.

Although the persistence of the administered *Lactobacillus *strains was not substantial after feeding had stopped, other faecal LAB strains were recurrently cultivated at two or more time points from all 12 volunteers (Table 3). The RAPD fingerprinting strategy was able to detect the persistence of these strains within the faeces for greater than 28 days in several of the volunteers (Fig. 6). Reproducible fingerprints were obtained for *Lactobacillus *species, *Streptococcus *species, *Enterococcus *species, and *Weissella *species isolates that all persisted in this way (Table 2 and 3; Fig. 2 and 6). Several strains were also the dominant cultivable isolates recovered from the faeces of certain volunteers, suggesting that they were colonising that individual's gut. For example, the *Enterococcus sanguinicola *strain (RAPD type 39, representative isolate G-02-a, Table 2; Fig. 2) recovered from volunteer G was first isolated at 14 days prior to commencing the feeding study and the same strain was also cultivated from their faeces at each subsequent sampling point until day 21 (see Fig. 6 for day 0 and day 21 RAPD fingerprints). At the -14 day sampling point this enterococcal strain was estimated to represent 1% of the cultivable diversity (1.8 × 10^4 ^cfu per g faeces), however, within day 0 and day 6 samples it represented 99% of the observed growth (approximately 1.75 × 10^5 ^cfu per g faeces); at day 21 it still represented 88% of the cultivable diversity, however, on day 28 it was not detected.

All the volunteers were colonised with persistent LAB strains (specific to each individual) that represented greater than 1% of their viable faecal growth; at least one of these strains was identified to the species level for each volunteer except J (Table 3). Apart from sharing of the *L. salivarius *NCIMB 30211 and *L. acidophilus *NCIMB 30156 strains present within the administered feeding capsule, only one other strain was detected in two volunteers, the *L. rhamnosus *RAPD type 41 strain (Table 2). This *L. rhamnosus *strain was shared by individuals P and T (Table 2 and Table 3). Overall, these results demonstrate the ability of the fingerprinting strategy to detect and track the population biology of cultivable faecal strains representative of a broad range of LAB species.

## Discussion

We successfully developed a rapid, colony-based strain typing strategy that was able to track two *Lactobacillus *strains from feeding via a capsule through to faecal discharge in human volunteers. The RAPD typing system was capable of genotyping a wide variety of LAB species and its efficacy on single colonies provided a means to rapidly discriminate LAB isolates cultivated from human faeces. Evidence for survival and growth of the *L. salivarius *strain was most convincing as it was not detected in any of volunteers prior to the feeding study (Table 3). In contrast, the *L. acidophilus *strain used in the capsule represented a very common genotype used in commercial applications (Table 2). Hence the appearance of *L. acidophilus *isolates which matched the feeding strain NCIMB 30156 may have been less attributable to consumption of the capsule. However, statistical analysis demonstrated that the distribution of *L. acidophilus *NCIMB 30156 after the feeding trial was significant in terms of the number of positive volunteers and in the majority of these positive individuals it was the dominant cultivable LAB strain in faeces.

As far as we are aware, previous studies evaluating the dynamics of LAB consumption by humans have not examined the cultivable faecal diversity at the strain level. Several studies have used cultivation-independent methods such as real-time PCR to quantify the DNA from probiotic strains present in faeces by extrapolating this amplification data to estimate of the numbers of bacteria. Bartosch et al. [18] used real-time PCR to estimate the total numbers of *Bifidobacterium *species present in the faeces of elderly people taking a probiotic containing two *Bifidobacterium *strains and an inulin-based prebiotic. They demonstrated that probiotic consumption increased the overall size of *Bifidobacterium *population in their subjects as estimated by increase yields in the species-specific PCR and also used cultivation-based approaches to show that more LAB species were present in the probiotic consuming subjects.

Maruo et al. [19] used RAPD to identify a strain-specific marker for the probiotic strain *Lactobacillus lactis *subsp. *cremoris *FC, and used real-time PCR to detect the strain's DNA within the faeces of human subjects taking the probiotic. They were able to show that the strain's DNA persisted during probiotic administration suggesting that between 10^5 ^and 10^9 ^bacterial cells were present per g of faeces. However, no cultivation and detection of the *L. lactis *subsp. *cremoris *strain FC was performed on the faecal samples [19] to indicate that the strain remained viable and actively colonised the gut during probiotic administration. Real-time PCR is a highly sensitive method, however, its dependence on detecting DNA and the fact that minute traces of DNA may take longer than cells to be completely cleared from the digestive tract, means that the method can be misleading in terms of providing functional information on the viability and persistence of an administered probiotic.

We have also shown that many commercial marketed probiotic products contain the same LAB strain (Table 2). Our RAPD typing was able to cluster genetically identical strains such as the multiple isolates matching the *L. acidophilus *Type strain (LMG 9433^T^; RAPD type 1), *L. casei *Type strain (LMG 6904^T^; RAPD type 10) and commonly used *L. rhamnosus *strains (MW and FMD T2; RAPD type 20). Studies by Yeung et al. [6] and Vancanneyt et al. [7] have also shown that multiple probiotic products often contain common LAB strain types. The fingerprinting method was also highly discriminatory distinguishing closely related taxa within the *L. casei *group (Fig. 2), yet at the strain level detecting 9 types among the 11 isolates examined from this group. The RAPD PCR-fingerprinting method also proved very robust and reproducible, with reference strains and cultivated faecal strains producing exactly the same amplified polymorphisms at widely disparate sampling and analysis points (see Fig. 2 and Fig. 6). This reproducibility and the amenability of PCR-fingerprinting to high throughput analysis enabled it to be used to examine the molecular epidemiology of *Lactobacillus *consumption by humans for the first time.

Our analysis demonstrated that for the *Lactobacillus *strains administered in the feeding study, long term persistence after consumption was not observed. Interestingly, persistence for greater than 21 days was only observed in volunteer S, the oldest subject in the study (age 65), from which the *L. salivarius *NCIMB 30211 capsule strain was recovered up to day 28 of the study. Increased probiotic colonisation in older people has been observed by others [18] and it will be intriguing to examine this phenomenon further using the colony fingerprinting method. The persistence seen with the subject-specific LAB strains cultivated from faeces is also interesting in this regard. Commercialisation of LAB strains for probiotic use is dependent on a number of factors, however, from our study and other work, it appears that many commercialised LAB strains are genotypically identical to reference strains deposited in recognised culture collections (Table 2). The fingerprinting strategy described herein could be used to select LAB strains with better persistence in human populations by screening a large population of healthy people, and selecting the dominant LAB strain types for evaluation as probiotics.

## Conclusion

We have shown that specific *Lactobacillus *strains consumed as part of a feeding study can be tracked through gastrointestinal passage via a colony-based strain typing strategy. The ability to identify specific LAB strains in faeces after human consumption provides a means to answer many important questions concerning the clinical use of probiotics. Our fingerprinting strategy could be used to identify the presence of the LAB isolates of the same genotype as potential probiotics prior to their administration in clinical trials, therefore allowing outcome measures dependent on the probiotic to be distinguished from those dependent on individuals which may naturally carry the same LAB strain. Overall, the successful application of molecular epidemiological techniques to cultivable bacterial populations within the human gut provides a platform for future systematic studies on the development of probiotics, as well as a rapid means to assess the strain diversity in healthy versus diseased humans.

## Methods

### Bacterial strains and cultivation

*Lactobacillus *reference strains were obtained from the Belgium Coordinated Collections of Microorganisms (BCCM; http://bccm.belspo.be/). Additional commercial LAB isolates were obtained from Cultech Ltd (Port Talbot, Wales, UK) or cultured directly from commercially marketed probiotic products as described below; a list of the strains used in this study is shown in Table 2. All strains of LAB were cultivated on MRS agar or in MRS broth (Oxoid, Basingstoke, UK) for 24 to 72 hours at 37°C. Commercial probiotic capsules and powders were resuspended in 5 ml MRS broth and serial dilutions plated onto MRS agar. To improve the isolation of LAB species from faecal samples, the semi-selective capacity of MRS agar was enhanced by the additional of 120 units per ml of Polymixin B (MRS-P medium; Polymixin B from, Sigma-Aldrich, Gillingham, UK). Fresh growth of purified faecal isolates was swabbed and resuspended in MRS broth containing 8% vol/vol dimethylsulphoxide prior to storage at -80°C. Frozen strains were revived by swabbing the surface of the frozen resuspension and plating onto MRS agar followed by incubation as above.

### RAPD PCR fingerprinting

For standard RAPD fingerprinting, DNA was extracted from 5 ml overnight broth cultures of LAB as previously described [13]. For the rapid fingerprinting protocol, preparation of DNA from single colonies was carried out as follows. A sterile 200 μl plastic pipette tip was inserted into a single freshly grown (no longer that 72 hours of plate growth) bacterial colony, resuspended into 50 μl of sterile 5% Chelex^® ^100 resin solution (Sigma-Aldrich, Gillingham, UK), and then plated onto MRS agar to provide a pure reference culture. The DNA extraction tubes were stored frozen at -20°C prior to the extraction of DNA for PCR. After thawing, the samples were boiled for 5 min and immediately placed on ice for a further 5 min; this heating and cooling cycle was repeated once to extract DNA. The resin was removed by brief centrifugation and 2 μl of the clear supernatant DNA solution used for the RAPD PCR.

PCR fingerprinting was carried out using a procedure that was modified from that described [13]. RAPD primers 201 to 300 (10 μg aliquots) were purchased from the Nucleic Acid Protein Service Unit at the University of British Columbia, Vancouver, Canada http://www.michaelsmith.ubc.ca/services/NAPS/. The primers that were found to be appropriate for LAB typing (272, 277 and 287; Table 1) were subsequently ordered individually in bulk from MWG Biotech (Covent Garden, London), dissolved as stocks in water at 100 pmol/μl and stored frozen. All PCR reagents were purchased from Qiagen Ltd. (Crawley, UK) and routine fingerprinting was carried out in a 25 μl reaction mixture containing: 2.5 μl PCR buffer, 5 μl Q-solution, 1.5 μl 25 mM MgCl_2 _(3 mM final concentration), 0.5 μl 10 mM dNTPs mixture (200 μM final concentration), 4 μl of 10 pmol/μl stock of RAPD primer, 2 μl of template DNA (approximately 40 ng) and 0.2 μl (1 unit) of Taq DNA polymerase. The PCR thermal cycles were carried out on a Flexigene Thermal Cycler (Techne Ltd., Newcastle, United Kingdom) as follows (ramping time between temperatures): (i) 4 cycles of 94°C for 5 min., 36°C for 5 min. (70 sec. cooling time), and 72°C for 5 min. (70 sec. heating time), (ii) 30 cycles of 94°C for1 min. (55 sec. to heat from 72°C), 36°C for 1 min. (60 sec to cool), 72°C for 2 min. (70 sec. to heat); and (iii) a final extension of 72°C for 6 min. followed by a hold at 4°C indefinitely.

All reference LAB strains (Table 2) were typed in duplicate and the type strain *L. acidophilus *LMG 9433^T ^was also used as an internal reproducibility control throughout all RAPD analysis, with multiple repeats performed to ensure RAPD typing was reproducible. Fingerprint profiles were separated by standard gel electrophoresis [13] using 1.5% high resolution agarose gels (Sigma-Aldrich, Poole UK). RAPD fingerprints were analysed using computer software (Gel Compar II, Appied Maths, Sint-Martens-Latem, Belgium) and fingerprint profiles compared by calculation of the Dice coefficient and clustering using the unweighted pair-group method average (UPGMA); isolates with RAPD fingerprint Dice coefficients greater than 0.85 were designated as a distinct bacterial strain.

### Molecular systematics

The 16S rRNA gene was used as the primary means to identify LAB isolates and other bacteria isolated during the feeding study. The primers applied by Yeung et al. [16] PAF, 5'-AGA GTT TGA TCC TGG CTC AG-3' and 536-R, 5'-GTA TTA CCG CGG CTG CTG-3', were used to amplify a 528 bp portion of the 16S rRNA gene. The resulting PCR product was sequenced on both strands using the latter primers and Applied Biosystems Big Dye Terminator ready reaction mix version 3.1, with subsequent analysis on an Applied Biosystems ABI-Prism 3100 automated sequencer. The end sequence reads were aligned, error checked and trimmed to 500 nucleotides to produce a consensus sequences using BioEdit [20]. Sequences were compared to: (i) the Ribosomal Database Project II (RDP II; http://rdp.cme.msu.edu/) using the sequence match tool, and (ii) GenBank using the Basic Local Alignment Search Tool (BLAST) at the National Centre for Biotechnological Information (NCBI; http://www.ncbi.nlm.nih.gov/), to facilitate identification.

To further enable accurate speciation within the genus *Lactobacillus*, 116 full-length 16S rRNA genes for reference isolates and type strains within this group were downloaded from the RDP II site and trimmed to match the 500 nucleotide portion obtained from isolates as above. The sequences were aligned using CLUSTAL W [21] and analysed phylogenetically using MEGA 3.1. Several tree-construction algorithms were evaluated; genetic distance trees drawn using the Jukes-Cantor neighbour-joining method were selected for the study because they produced phylogenies that were congruent with the current LAB taxonomy of LAB. To confirm identification of novel non-*Lactobacillus *species isolated during the study, 16S rRNA genes from their closest RDP II match (species Type strains) were included in the phylogenetic analysis. A total of 54 partial 16S rRNA gene sequences were determined as part of this study and have been deposited in GenBank (Accession numbers are shown in Table 2).

### *Lactobacillus *feeding study

A probiotic-like capsule (manufactured by Cultech Ltd, Port Talbot, UK) containing the following strains was formulated according to standard food product guidelines: *L. salivarius *strain NCIMB 30211 and *L. acidophilus *strain NCIMB 30156. The two strains were selected merely on the basis that each had been previously used in probiotic formulations manufactured by Cultech Ltd. The probiotic capsule was taken once a day for 14 days during feeding study. Fifteen healthy volunteers were initially enrolled and 12 participated in the final study. All volunteers gave written consent to provide faecal samples and take the *Lactobacillus *capsules as part of the feeding trial; all were free to withdraw from the study at any point. In addition, no exclusion criteria applied to the volunteers and they were free to eat normally (including diary products) or take medicinal drugs (such as antibiotics) at any point in the study.

Faecal samples were provided as follows: (i) Day -14, 2 weeks prior to commencing probiotic administration as a pre-study control; (ii) Day 0, the start day for probiotic feeding with the fecal sample taken before ingestion of the first capsule; (iii) Day 2; (iv) Day 7; (v) Day 14 as the last day the probiotic formulation was taken; (vi) Day 21, 21 days after probiotic consumption and 7 days following cessation of feeding; and finally (vi) Day 28, 28 days after first probiotic consumption and 14 days following cessation of probiotic administration. Ethical approval for the feeding study was granted by Cardiff School of Biosciences, Cardiff University (Approval number 079-1).

### Cultivation of LAB from faecal samples

Fresh faecal samples were weighed, diluted 1:10 MRD diluent (Oxoid, Basingstoke, UK) containing 15% glycerol, and frozen at -80°C; no significant loss of cultivable diversity or viability was observed when freshly resuspended and plated faecal samples were compared to replicate samples that had been stored frozen. Serial dilutions were plated in replicate onto MRS and MRS-P agar, incubated at 37°C for 72 hours, and enumerated quantitatively and qualitatively prior to random picking of up to 10 the different colony morphotypes for RAPD fingerprinting. Each serial dilution plate was documented using digital photography; if RAPD detected the presence of either feeding study strain (*L. salivarius *strain NCIMB 30211 or *L. acidophilus *strain NCIMB 30156; see Fig. 6), then retrospective counting of all the morphotypes associated with the strains was performed to determine a total count per gram of faeces.

### Statistical analysis

For enumeration of the faecal counts on MRS-P agar, the mean and standard error of the mean were determined and a 2-sample t-test to compare means (all numerical analysis was performed using MINITAB^® ^Release 15, Minitab Inc.). The overall results of the *Lactobacillus *feeding study were analysed non-parametrically using Chi square because of the limited number of subjects and the variables measured. A 2 × 2 data table was constructed for the analysis categorising the data as follows: two columns for the number of volunteers positive and negative for the administered *Lactobacillus *strains, respectively, and two rows for before and after capsule consumption, respectively (positive cultures for any given volunteer were only counted once).

## Authors' contributions

EM and AM developed the strain typing methods, with SP providing several of the LAB strain for analysis. EM, AM, SP, and IG planned the feeding study. PD carried out the computer aided comparison of strain fingerprints. EM wrote the manuscript. All other authors contributed towards the drafting of paper, have read and approved the final manuscript.
